# NADPH-Thioredoxin Reductase C Mediates the Response to Oxidative Stress and Thermotolerance in the Cyanobacterium *Anabaena* sp. PCC7120

**DOI:** 10.3389/fmicb.2016.01283

**Published:** 2016-08-18

**Authors:** Ana M. Sánchez-Riego, Alejandro Mata-Cabana, Carla V. Galmozzi, Francisco J. Florencio

**Affiliations:** Instituto de Bioquímica Vegetal y Fotosíntesis, Universidad de Sevilla-Consejo Superior de Investigaciones CientíficasSeville, Spain

**Keywords:** *Anabaena*, thioredoxin reductase, peroxiredoxin, oxidative stress, cyanobacteria

## Abstract

NADPH-thioredoxin reductase C (NTRC) is a bimodular enzyme composed of an NADPH-thioredoxin reductase and a thiioredoxin domain extension in the same protein. In plants, NTRC has been described to be involved in the protection of the chloroplast against oxidative stress damage through reduction of the 2-Cys peroxiredoxin (2-Cys Prx) as well as through other functions related to redox enzyme regulation. In cyanobacteria, the *Anabaena* NTRC has been characterized *in vitro*, however, nothing was known about its *in vivo* function. In order to study that, we have generated the first knockout mutant strain (ΔntrC), apart from the previously described in *Arabidopsis*. Detailed characterization of this strain reveals a differential sensitivity to oxidative stress treatments with respect to the wild-type *Anabaena* strain, including a higher level of ROS (reactive oxygen species) in normal growth conditions. In the mutant strain, different oxidative stress treatments such as hydrogen peroxide, methyl-viologen or high light irradiance provoke an increase in the expression of genes related to ROS detoxification, including AnNTRC and peroxiredoxin genes, with a concomitant increase in the amount of AnNTRC and 2-Cys Prx. Moreover, the role of AnNTRC in the antioxidant response is confirmed by the observation of a pronounced overoxidation of the 2-Cys Prx and a time-delay recovery of the reduced form of this protein upon oxidative stress treatments. Our results suggest the participation of this enzyme in the peroxide detoxification in *Anabaena*. In addition, we describe the role of *Anabaena* NTRC in thermotolerance, by the appearance of high molecular mass AnNTRC complexes, showing that the mutant strain is more sensitive to high temperature treatments.

## Introduction

Cyanobacteria and chloroplasts possess redox systems that allow them to link the photosynthetic electron transport process with metabolism regulation through disulphide-mediated thiol-based redox mechanisms. These systems are also important to cope with ROS generation derived from molecular oxygen production (Lindahl et al., 2011; Balsera et al., 2014). Thioredoxin (Trx) is a class of small redox proteins that play a main role in metabolic regulation, by regulating the activity of different enzymes via disulphide-dithiol exchange receiving electrons from the photosynthesis reaction, through the FTR, and from the NADPH, through the NTR (Florencio et al., 2006; Balsera et al., 2014). Prxs are thioredoxin-dependent peroxidases, which constitute the link between redox regulation and ROS detoxification (Dietz, 2007, 2011). The Trx system has been proposed to be a key regulatory mechanism for many proteins and metabolic pathways in photosynthetic organisms (Lindahl and Florencio, 2003; Pérez-Pérez et al., 2006; Mata-Cabana et al., 2007, 2012; Montrichard et al., 2009; Lindahl et al., 2011; Balsera et al., 2014).

Analogously to what was described for *Mycobacterium leprae* (Wieles et al., 1995), a new type of NTR fused to a Trx domain was identified in the chloroplast of rice and *Arabidopsis thaliana* (Serrato et al., 2004). This new NTR was named NADPH-thioredoxin reductase C (NTRC). In plants, NTRC has been described to be involved in the protection of the chloroplast against oxidative stress damage through effective reduction of 2-Cys Prx (Moon et al., 2006; Pérez-Ruiz et al., 2006). Although the characteristic enzymatic activities have been measured independently for both domains, NTR and Trx (Moon et al., 2006), it has been shown that the NTR domain is not an effective reductant for other chloroplast Trxs, being the Trx domain necessary for reduction of its target proteins (Serrato et al., 2004; Bohrer et al., 2012; Lee et al., 2012; Balsera et al., 2014). Besides its role in chloroplasts, NTRC is also important for the redox regulation in non-photosynthetic plastids (Cejudo et al., 2012; Kirchsteiger et al., 2012). Additionally, NTRC is involved in the regulation of other processes like starch synthesis or tetrapyrroles metabolism (Michalska et al., 2009; Richter et al., 2013; Balsera et al., 2014; Pérez-Ruiz et al., 2014). Recently, heat shock-regulated chaperone activity has been demonstrated for the *Arabidopsis* NTRC (Chae et al., 2013) in which a switch in the oligomerization state of the protein occurs from low to high molecular mass complexes.

A close phylogenetic relationship has been reported between the plant and cyanobacterial NTRCs, suggesting a cyanobacterial origin for the plant enzyme, whose gene was transferred to the eukaryotic genome during the chloroplast evolution (Florencio et al., 2006; Pascual et al., 2010, 2011; Balsera et al., 2014). However, not all cyanobacteria possess an *ntrc* coding gene (Florencio et al., 2006; Pascual et al., 2010; Balsera et al., 2014). Interestingly, two different antioxidant strategies to cope with peroxides were described in cyanobacteria (Pascual et al., 2010). One of the strategies, described for *Synechocystis* sp. PCC 6803 (hereafter *Synechocystis*), consists of a high peroxidase/catalase activity. In the case of *Anabaena* sp. PCC 7120 (hereafter *Anabaena*), the peroxide detoxification system is formed by AnNTRC, 2-Cys Prx and sulfiredoxin. The 2-Cys Prx from *Synechocystis* is more resistant to overoxidation, but since this cyanobacterium lacks the NTRC and sulfiredoxin enzymes such overoxidation is irreversible. In contrast, the *Anabaena* 2-Cys Prx is more sensitive to overoxidation, but it can be reversed by the joint activity of the AnNTRC and sulfiredoxin (Pascual et al., 2010; Boileau et al., 2011), indicating that the *Anabaena* system is more similar to which is found in plant chloroplasts. A biochemical characterization of the *Anabaena* NTRC together with a comparative analysis of the plant enzyme confirmed that the *Anabaena* enzyme is similar to the plant type in its bimodular nature and in its enzyme activities, including the capacity to reduce 2-Cys Prxs from *Anabaena* and rice. However, this was not true for the *Synechocystis* Prx, supporting the idea of the two evolutionary divergent antioxidant strategies (Pascual et al., 2011). Additionally, the cyanobacterial NTRC was able to partially rescue the *ntrC Arabidopsis* mutant, pointing out the evolutionary relation between both *Anabaena* and chloroplast enzymes (Pascual et al., 2011).

Even though the *in vitro* features of the *Anabaena* NTRC have been analyzed, nothing is known about its role in a living cyanobacterium. With this aim we have generated an *Anabaena* mutant strain lacking the AnNTRC protein and we have examined its function upon different stress conditions allowing us to determine its role as a main component of the oxidative stress resistant system in cyanobacteria lacking the catalase/peroxidase module.

## Materials and Methods

### Bacterial Strains, Plasmids, and Growth Conditions

*Anabaena* cells were grown photoautotrophically in liquid BG11 (Rippka et al., 1979) at 30°C under continuous illumination (50 μE m^-2^ s^-1^) and bubbled with a stream of 1% (v/v) CO_2_ in air. Experiments were performed using cultures from the mid-logarithmic phase (3–5 μg chlorophyll mL^-1^) cultivated without antibiotics. To analyze the effects of high light intensities, *Anabaena* strains were grown until the exponential phase, diluted to 1 μg chlorophyll mL-1 and shifted to high light intensity under 500 μE m^-2^ s^-1^, and the temperature was kept at 30°C by applying a 5-cm-thick water filter. For heat shock conditions, cells were grown in a transparent water bath at 45–50°C. For the H_2_O_2_ treatment, WT and ΔntrC strains were grown until the exponential phase, diluted to 5 μg chlorophyll mL-1 and then, 0,1 mM or 0.4 mM H_2_O_2_ was added. For the treatment with methyl viologen, both strains were grown until the exponential phase, diluted to 3 μg chlorophyll mL-1 and 0,5 μM of the chemical was added. The treatments conditions were optimized for *Anabaena* in our laboratory to better visualize the difference between the WT and ΔntrC strains. Cyanobacterial growth was monitored by measuring the absorbance at 750 nm (OD 750 nm) and chlorophyll content.

The *ntrC* knock-out mutant (strain ΔntrC) was generated by homologous recombination, replacing the *Anabaena ntrC* gene (*all0737*) with a spectinomycin cassette. DNA fragments upstream and downstream of the *ntrC* gene were amplified by a two-step PCR process using primer pairs NtrC_Up_F and NtrC_Up_R and NtrC_Down_F and NtrC_Down_R, respectively (Supplementary Table S1). Both fragments were ligated by overlapping PCR and cloned into p278ΔNTRC. The spectinomycin resistance cassette, excised by digestion with BamHI from the plasmid pRL161, was inserted into the BamHI site between the upstream and downstream fragments. The generated plasmid was transferred to *Anabaena* by triparental mating with *Escherichia coli* strains HB101 (pCSMI61, pRL623) and ED8654 (pRL443) as previously described (Elhai and Wolk, 1988). The *Anabaena* recombinant clones were selected on BG11 supplemented with spectinomycin at 20 g/ml. Double recombinants were selected on the basis of their ability to grow in the presence of sucrose. Complete segregation of the mutation was confirmed by PCR.

### RNA Isolation and Northern Blot Analysis

Total RNA was isolated from 30 mL samples of *Anabaena* cultures in the mid-exponential growth phase (3–5 μg chlorophyll mL^-1^). Extractions were performed by vortexing cells in the presence of phenol-chloroform and acid-washed baked glass beads (0,25–0,3 mm diameter) as previously described (García-Domínguez and Florencio, 1997). Five micrograms of total RNA was loaded per lane and electrophoresed in 1,2% agarose denaturing formaldehyde gels and transferred to nylon membranes (Hybond N-Plus; Amersham, GE Healthcare, Buckinghamshire, England). Prehybridization, hybridization, and washes were in accordance with Amersham instruction manuals. All probes were synthesized by PCR and oligonucleotide pairs used are described in (Supplementary Table S1). All filters were stripped and re-hybridized with the constitutively expressed *rnpB* gene from *Anabaena*. Hybridization signals were quantified with a Cyclone Phosphor System (Packard, Meriden, CT, US). The histograms depicted in the **Figures 3D, 4D** and **5D** correspond to relative mRNA levels, calculated by quantifying the radioactive signals and normalizing them to *rnpB* signal. The data showed represent the average of three independent experiments.

### Western Blot Analysis

For Western blot analysis, 5 μg of total protein from soluble extracts were separated by 12% acrylamide SDS-PAGE gels, transferred to nitrocellulose membranes (Bio-Rad), and probed with AnNTRC (1:1000), 2-Cys Prx (1:5000) and TrxA (thioredoxin A) (1:3000) antibodies. In order to detect High Molecular Mass (HMM) species 10% acrylamide non-reducing SDS-PAGE (where reducing agents were avoided in the loading buffer) and native-PAGE were used. Signals were detected using an anti-rabbit secondary antibody (Sigma-Aldrich, St Louis, MO, US) and the ECL-Plus immunoblotting detection system (GE Healthcare, Buckinghamshire, England).

### AnNTRC Activity Determination

AnNTRC activity was determined by the reduction of DTNB according to the method described by Holmgren and Björnstedt (1995). The reaction was performed in 100 mM potassium phosphate buffer, pH 7.0, 2 mM EDTA, 5 mM DTNB, 150 μM NADPH, and 50 μg *Anabaena* soluble extracts in a total volume of 1 ml. The reduction of DTNB was monitored by the increase in OD 412 nm. Assays were performed at least three times.

### *In vivo* Detection of ROS Production Using CM-H2DCFDA

ROS level from *Anabaena* cultures in the mid-exponential growth phase (3–5 μg chlorophyll mL^-1^) were examined by CM-H2DCFDA fluorescence (Rastogi et al., 2010; Boileau et al., 2011). The cells were harvested by centrifugation and immediately resuspended in a buffer containing 25 mM Hepes-NaOH (pH 7,6) and 35 μM CM-H2DCFDA (Molecular Probes^®^, Invitrogen Life Technologies^TM^, Grand Island, NY, US). The oxidized form of CM-H2DCFDA emits fluorescence in the 520 nm region when excited with light at a 494 nm wavelength. After incubation in this buffer for 15 min in the dark, the cells were centrifuged and resuspended in fresh buffer. Fluorescence was monitored with a confocal microscope Leica TCS SP2 (Leica Microsystems, Mannheim, Germany).

### Size-Exclusion Chromatography (SEC)

The AnNTRC complexes were analyzed by SEC using an ÄKTA FPLC (Amersham, GE Healthcare, Buckinghamshire, England) with a Superdex 200 HR 10/30 column equilibrated with a 50 mM KH_2_PO_4_ (pH 7.1) buffer containing 150 mM NaCl at a flow rate of 0.5 ml min^-1^. Total protein extracts were prepared in 50 mM KH_2_PO_4_ (pH 7.1), 150 mM NaCl and 1 mM PMSF by bead beating using 0,1 mm glass beads. Two hundred micrograms of total protein from soluble extracts were loaded onto the column.

## Results

### AnNTRC Is Not Essential under Standard Growth Conditions

**FIGURE 1:**
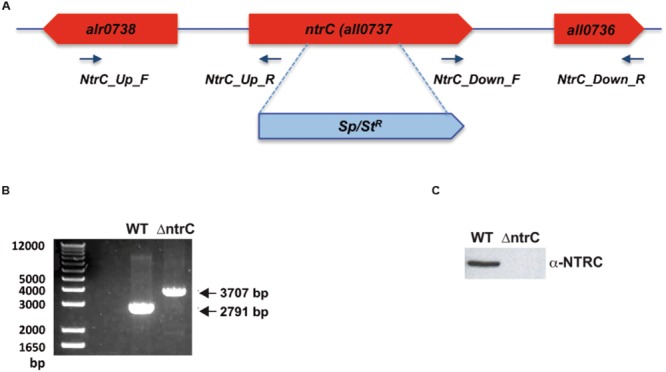
**ΔntrC mutant construction. (A)** *ntrC* locus representation, where the *ntrC* gene disruption and the spectinomycin marker gene insertion are depicted. The location of the primers used for the mutagenesis is also shown. **(B)** Confirmation of the mutant by PCR. The amplification of the WT sequence was 2791 bp, meanwhile the sequence after insertion was 3707 bp long. **(C)** Western blot showing the absence of AnNTRC in the mutant.

**FIGURE 2:**
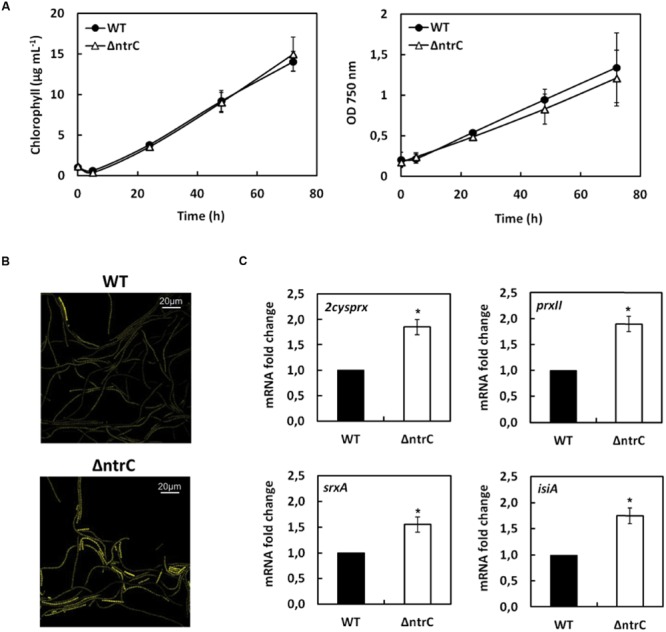
**AnNTRC is not essential under standard growth conditions. (A)** Graphic representation of growth of *Anabaena* WT and ΔntrC mutant strains under standard growth conditions. **(B)** Fluorescence micrographs of *Anabaena* where DCFH-DA probe was used to detect ROS. **(C)** Quantification of relative mRNA levels of *2cysprx, prxII, srxA* and *isiA* genes under standard growth conditions. Error bars show SE (*n* = 3). ^∗^ indicates significant differences at *P* < 0.01 compared with the corresponding wild type.

Although the biochemical properties of the NTRC of *Anabaena* have been studied *in vitro* in comparison with the rice enzyme (Pascual et al., 2011) its function and involvement in redox regulation *in vivo* has not been addressed yet. With this aim the ΔntrC knock-out mutant strain was generated in *Anabaena* by partial deletion of the *all0737* gene and insertion of a spectinomycin/streptomycin resistance marker (**Figures 1A,B**). This mutation resulted in the lack of the AnNTRC protein (**Figure 1C**). Under standard growth conditions (BG11C at 30C under 50 μE m^-2^ s^-1^ of continuous illumination) the ΔntrC mutant shows a normal growth compared to the WT strain (**Figure 2A**), revealing that this enzyme is not essential under these conditions. However, when using the fluorescent ROS-detecting probe DCFH-DA, an accumulation of ROS was detected in the ΔntrC mutant (**Figure 2B**), indicating an increase in ROS production or impairment in their removal. Additionally, Northern blot experiments showed an increased expression of the *2cysprx, prxII, srxA*, and *isiA* genes in the mutant strain (**Figure 2C**). The expression of these genes responds to oxidative stress. In this case, the increase is never more than 2 folds up, indicating an increment of intracellular ROS in the mutant, but never compared with the values obtained after any of the stress conditions applied. To further investigate the ΔntrC mutant response to oxidative stress this strain was subjected to different oxidative conditions such as H_2_O_2_, MV, and HL treatments.

### AnNTRC Is Involved in Antioxidant Response to H_2_O_2_ Treatment

**FIGURE 3:**
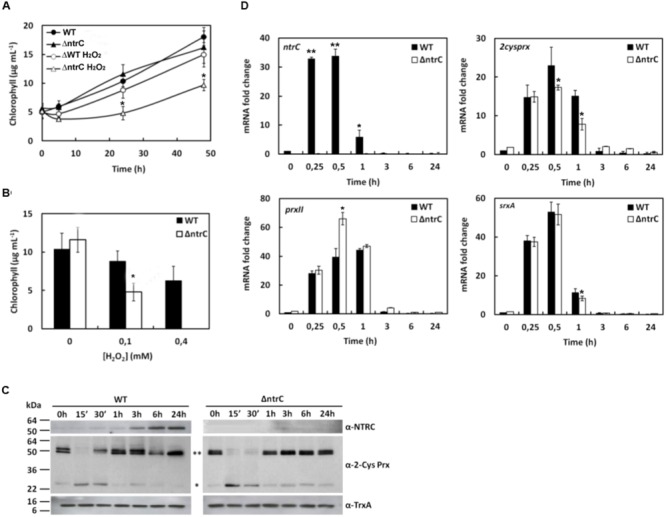
***Anabaena* ΔntrC mutant is sensitive to H_2_O_2_ addition. (A)** Growth of WT and ΔntrC strains after H_2_O_2_ addition. **(B)** Histogram representation of chlorophyll content at 24 h of WT and ΔntrC strains growth under 0,1 mM and 0,4 mM H_2_O_2_ treatments. **(C)** Western blot analysis of AnNTRC and 2-Cys Prx levels in response to 0,1 mM H_2_O_2_ addition. TrxA was used a loading control. ^∗∗^ Dimer. ^∗^ Monomer. **(D)** Quantification of relative mRNA levels of *ntrC, 2cysprx, prxII* and *srxA* genes in response to H_2_O_2_ addition. Error bars show SE (*n* = 3). Asterisks indicate significant differences (^∗^*P* < 0.05 and ^∗∗^*P* < 0.01) compared with the corresponding wild type.

The ΔntrC mutant displayed a higher sensitivity to 0,1 mM H_2_O_2_ treatment compared to the WT strain (**Figure 3A**). Moreover, 0,4 mM H_2_O_2_ turned out to be a lethal concentration after 24 h treatment for the mutant but not for the WT (**Figure 3B**). In addition, the amount of AnNTRC protein and the expression of the corresponding gene increased upon this condition (**Figures 3C,D**), suggesting a role for this protein in the antioxidant response. The plant NTRC has been demonstrated to be involved in H_2_O_2_ detoxification via 2-Cys Prx reduction (Pérez-Ruiz et al., 2006), what could suggest a similar detoxification mechanism involving NTRC for this cyanobacterium. Among the two different strategies found in cyanobacteria to cope with hydrogen peroxide (Pascual et al., 2010) *Anabaena* possesses the same as plant chloroplasts being equipped with NTRC, sulfirredoxin (Srx) and a 2-Cys Prx sensitive to overoxidation (Pascual et al., 2010). For instance, the AnNTRC was shown to efficiently reduce 2-Cys Prx from *Anabaena* and rice *in vitro* (Pascual et al., 2011). Therefore, to further investigate this activity *in vivo* and its role in peroxide reduction, the oxidation state of the *Anabaena* 2-Cys Prx was analyzed in cell lysates from the cyanobacterium upon H_2_O_2_ treatment. Under normal conditions 2-Cys Prx remains in its dimeric, oxidized form with the monomeric form being visible in the non-reducing SDS-PAGE mainly when it is overoxidized (Pascual et al., 2010). In this regard, the 2-Cys Prx overoxidation is more prolonged in the mutant than in the WT strain (**Figure 3C**), which can be explained by the inability to reduce Prx by AnNTRC, preventing the removal of H_2_O_2_, which will promote such overoxidation.

Besides *ntrC*, the expression of other genes was analyzed when treated with hydrogen peroxide. The *isiA* gene encodes an iron deficiency-related chlorophyll-binding protein (Michel and Pistorius, 2004) and, since its expression is known to be induced by hydrogen peroxide (Li et al., 2004; Singh and Sherman, 2007), it was used as a control. As expected, the expression of *isiA* increased under H_2_O_2_ treatment and this increase was similar for both WT and ΔntrC strains (Supplementary Figure S1). The induction response to hydrogen peroxide was also previously demonstrated for two peroxiredoxin genes, *2cysprx* and *prxII*, in the cyanobacterium *Synechocystis* (Pérez-Pérez et al., 2009) and for *2cysprx* in *Anabaena* (Yingping et al., 2014). Here we analyzed the expression of those genes in *Anabaena* and, as occurs in *Synechocystis*, both are induced by H_2_O_2_ treatment and this induction is independent of the presence of AnNTRC (**Figure 3D**). The *srxA* gene encodes a sulfiredoxin protein, homologous to the eukaryotic sulfiredoxins, which was shown to be able to reduce the sulfinic (SOOH) form of the 2-Cys Prx in *Anabaena*. Our results indicate that its gene expression level was induced under H_2_O_2_ treatment and it seems to be independent of AnNTRC (**Figure 3D**). The srxA gene was previously shown to be induced under oxidative stress conditions (Boileau et al., 2011). It must be mentioned here that the basal expression (0 h) for all these genes was higher in the mutant strain than in the WT (**Figures 2C** and **3D**) indicating that the ROS accumulation in the ΔntrC strain is high enough to influence gene expression (**Figure 2B**). However, this ROS content does not affect the 2-Cys Prx overoxidation (**Figure 3C**).

### AnNTRC Responds to the Oxidative Stress Generated by MV Treatment

**FIGURE 4:**
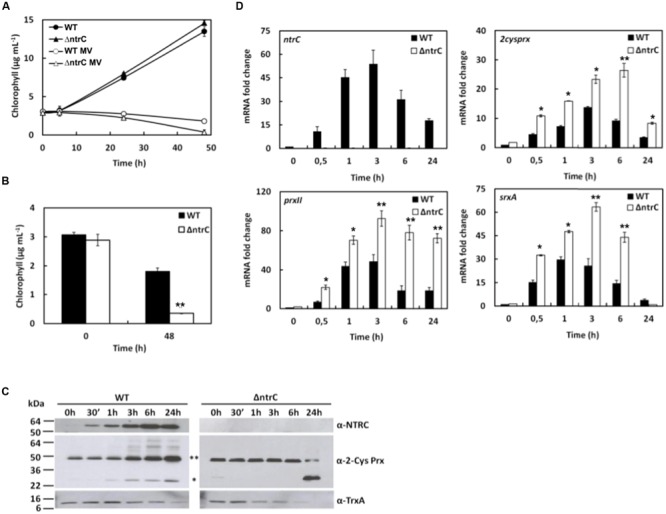
***Anabaena* ΔntrC mutant sensitivity to MV treatment. (A)** Growth of WT and ΔntrC strains after MV addition. **(B)** Histogram representation of chlorophyll content at 0 h and 48 h of growing of WT and ΔntrC strains under 0.5 μM MV treatment. **(C)** Western blot analysis of AnNTRC and 2-Cys Prx levels in response to MV. TrxA was used as loading control. ^∗∗^ Dimer. ^∗^ Monomer. **(D)** Quantification of relative mRNA levels of *ntrC, 2cysprx, prxII* and *srxA* and in response to MV addition. Error bars show SE (*n* = 3). Asterisks indicate significant differences (^∗^*P* < 0.05 and ^∗∗^*P* < 0.01) compared with the corresponding wild type.

In cyanobacteria the treatment with MV results in production of superoxide anion radicals, enhanced during oxygenic photosynthesis (Babbs et al., 1989; Krieger-Liszkay et al., 2011). This, in turn, leads to the formation of hydrogen peroxide and hydroxyl radicals mediated by the SOD activity. Thus, this treatment is expected to resemble the treatment with hydrogen peroxide but, probably, to a lesser extent since, in this case, the peroxide comes from the endogenous production, which is dependent on the photosynthesis electron transport as well as the SOD action. MV displays high toxicity and the ΔntrC mutant seems to be slightly more sensitive compared to WT (**Figures 4A,B**), probably due to the fact that AnNTRC would be only involved in the protection against the peroxides but not against the anion superoxide. The response of the genes to MV is similar to that obtained after hydrogen peroxide treatment, but delayed (**Figure 4D**). Furthermore, the induction of these genes seems to be higher and sustained in time in the mutant ΔntrC when it is compared to the WT strain. The 2-Cys Prx is highly overoxidized in the ΔntrC mutant only after 24 h of treatment, which is not observed in the WT (**Figure 4C**). Similarly to what is observed for the hydrogen peroxide treatment, both the *ntrC* gene and the AnNTRC protein increased by MV treatment (**Figures 4C,D**). It must be mentioned here that due to the high toxicity of the MV the cyanobacterium is dying and there is protein degradation, as it can be observed for the TrxA, used as loading control, but not for AnNTRC and 2-Cys Prx, which are needed for the antioxidant defense. The increase of the AnNTRC protein was also confirmed by determining the DTNB reductase activity. The AnNTRC capacity to reduce the substrate DTNB was measured after 4 h of MV treatment. As depicted in Supplementary Figure S2, the reduction of DTNB was much higher in the crude extract of MV treated cell than that measured for untreated cells, due to the higher accumulation of AnNTRC protein after the treatment. The ΔntrC mutant strain lacks this reductase activity (Supplementary Figure S2).

### AnNTRC Protects against the Oxidative Stress Generated by High Light Intensity Illumination

**FIGURE 5:**
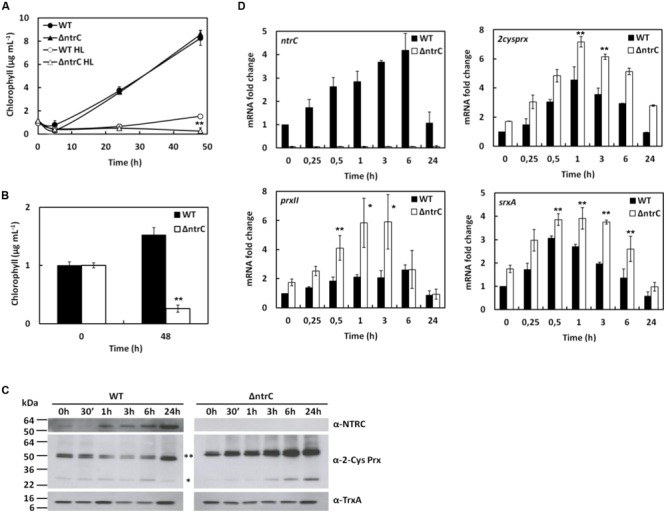
***Anabaena* ΔntrC mutant sensitivity to HL treatment. (A)** Growth of WT and ΔntrC strains after a shift to HL. **(B)** Histogram representation of chlorophyll content at 0 h and 48 h of growing of WT and ΔntrC strains under HL treatment. **(C)** Western blot analysis of AnNTRC and 2-Cys Prx levels in response to a shift to HL. TrxA was used as loading control. ^∗∗^ Dimer. ^∗^ Monomer. **(D)** Quantification of relative mRNA levels of *ntrC, 2cysprx, prxII* and *srxA* in response to a shift HL. Error bars show SE (*n* = 3). Asterisks indicate significant differences (^∗^*P* < 0.05 and ^∗∗^*P* < 0.01) compared with the corresponding wild type.

In the next step we tried a culture condition that generates endogenous oxidative stress by subjecting the cyanobacterium to a high light intensity (500 μE m^-2^ s^-1^). As in the case of the MV, the ROS production is dependent on photosynthetic electron transport, however, the driving action of the MV in such ROS generation is now missed. Also in this case the H_2_O_2_ production requires the SOD participation and the effect of the H_2_O_2_ is only partially responsible for the observed sensitivity. According to this, the sensitivity to high light displayed by the mutant strain is a little higher than that of the WT (**Figures 5A,B**). The H_2_O_2_ concentration generated does not seem to be enough to cause 2-Cys Prx overoxidation, as observed in the previous treatments (**Figure 5C**). However, it is high enough to increase the AnNTRC protein level (**Figure 5C**). Similarly, the expression of the tested genes also increased but in a lower rate than observed for H_2_O_2_ and MV (**Figure 5D**).

Overall, our data suggest that AnNTRC responds to H_2_O_2_ and that it is involved in the peroxide protection system.

### AnNTRC Mediates Thermotolerance in *Anabaena*

**FIGURE 6:**
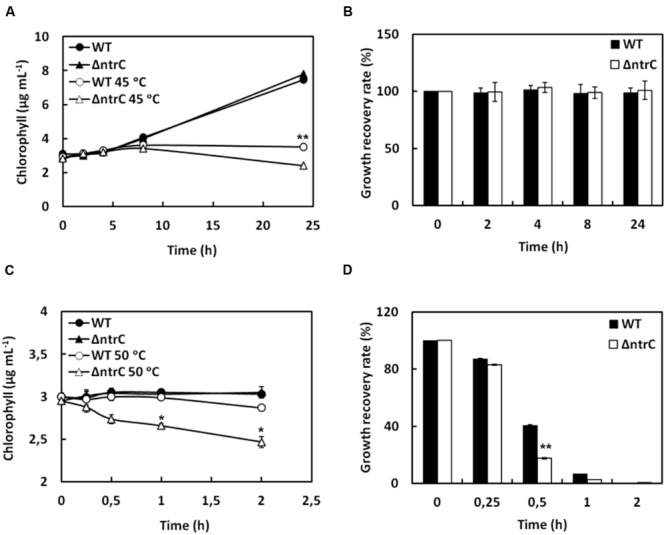
***Anabaena* ΔntrC mutant is sensitive to heat shock. (A,C)** Growth of WT and ΔntrC strains under high temperature conditions, 45°C **(A)** and 50°C **(C)**. Growth was monitored by measuring chlorophyll content. WT and ΔntrC strains were grown until the exponential phase, diluted to 3 μg chlorophyll mL^-1^ and then, the high temperature treatment was applied. **(B,D)** Histogram representation of the growth recovery rate at normal growth temperature (30°C) for WT and ΔntrC strains 24 h after heat shock treatment at 45°C **(B)** and 50°C **(D)**. Both strains were subjected to heat shock during the indicated time and the growth ratios after 24 h between treated and untreated cells were calculated. Error bars show SE (*n* = 3). Asterisks indicate significant differences (^∗^*P* < 0.05 and ^∗∗^*P* < 0.01) compared with the corresponding wild type.

Cyanobacteria as a group are ubiquitously distributed and are often found in extreme environmental conditions, being able to survive in extremes temperatures from 60 to 74°C. In *Anabaena*, the nitrogen fixation is sensitive to temperatures above 42°C and chaperones induction has been observed at temperatures upon 39–45°C in *Anabaena* sp. strain L-31 (Rajaram et al., 2014). It has been shown recently that NTRC is involved in thermotolerance in *Arabidopsis* (Chae et al., 2013). Plants overexpressing NTRC displayed an enhanced thermotolerance. It was also demonstrated that NTRC, in addition to its disulphide reductase activity, can form HMM complexes under high temperature conditions and can act as a chaperone (Chae et al., 2013). In a similar manner, we proceeded to explore for AnNTRC this new reported function. Firstly, we analyzed the survival of both the WT and ΔntrC strains at high temperatures, 45 and 50°C. 45°C is a more permissive temperature, which allowed us to measure the growth of the mutant and the WT strains during 24 h, period (**Figure 6A**). However, 50°C is a lethal temperature for *Anabaena* and we could only check until 2 h of growth (**Figure 6C**). In both cases, the mutant showed more sensitivity than the WT. In order to better evaluate the effect of high temperatures we analyzed the growth recovery rate after increasing times of high temperature treatments. At 45°C there are no remarkable differences (**Figure 6B** and Supplementary Figure S3A), but at 50°C we observed that WT strain recovered better than the mutant with the largest difference after 30 min at 50°C (**Figure 6D** and Supplementary Figure S3B).

**FIGURE 7:**
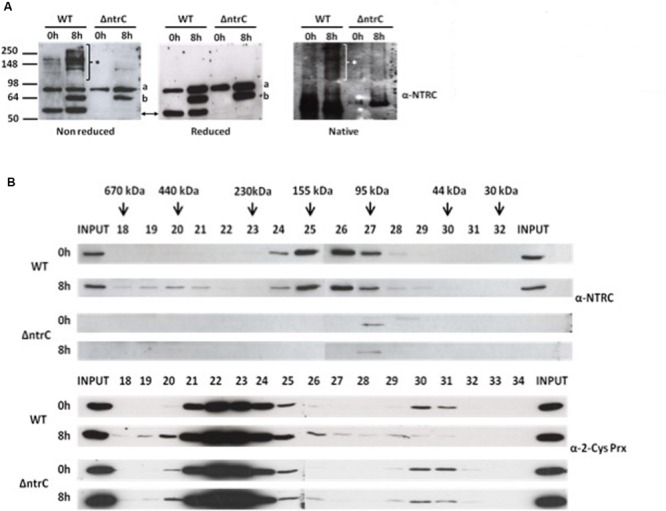
**Oligomerization of AnNTRC and 2-Cys Prx under heat shock. (A)** Non-reducing SDS-PAGE, reducing SDS-PAGE and native-PAGE Western blots of AnNTRC at 0 h and 8 h of treatment at 45°C. The AnNTRC monomer is pointed by arrows, the asterisk (^∗^) marks the localization of the oligomers and “a” and “b” indicates two unspecific bands. **(B)** Western blot of the different fractions obtained by SEC of protein extracts from WT and ΔntrC strains before and after 8 h of treatment at 45°C. The top panel corresponds to the Western blot against AnNTRC and the bottom one to the one against 2-Cys Prx. The fractions corresponding to the different protein molecular markers used to calibrate the column are indicated in the top blot.

These results suggest that AnNTRC is involved in thermotolerance in *Anabaena*. To further investigate this NTRC function in cyanobacteria we also analyzed the oligomerization state of AnNTRC after heat shock treatment. To do that, we incubated the *Anabaena* cells at 45°C and checked the AnNTRC oligomerization state after 8 h treatment by Western blot using non-reducing SDS-PAGE or native-PAGE (**Figure 7A**). In both cases, HMM AnNTRC species appeared, but under reducing conditions these HMM species were not detected (**Figure 7A**). This result was confirmed by SEC, which allowed us to separate the different AnNTRC species according to its molecular mass. Thus, in the untreated sample AnNTRC eluted between the fractions 24 to 27 (**Figure 7B**), which in molecular size would correspond to the dimeric form. However, when the cells were cultured at 45°C, AnNTRC also appeared in fractions from 18 to 21, which could correspond to HMM species in the range of 670 to 440 kDa (**Figure 7B**). As previously reported, HMM species of 2-Cys Prx also appeared after heat shock treatment (Morgan and Veal, 2007; Barranco-Medina et al., 2009; Dietz and Pfannschmidt, 2011). In our experiments, for the WT strain, the formation of these species correlates with the decrease of the dimer (**Figure 7B**). In contrast, in the mutant strain the HMM species appeared in lesser extent and a decrease of the dimer was not detected (**Figure 7B**). This result suggest that the higher oligomerization of 2-Cys Prx in response to 45°C temperature treatment is partially dependent on NTRC in *Anabaena*. Taking together these results suggest that the AnNTRC oligomerization is regulated by temperature and it may be one of the proteins involved in the thermotolerance response in cyanobacteria.

## Discussion

The thioredoxin reduction function in cyanobacteria is performed by the FTR and the NTR, receiving the reducing equivalents from the photosynthetic electron transport and the NADPH, respectively. The genome analysis of the cyanobacteria phylum revealed that, despite being photosynthetic organisms, not all species have genes coding for FTR, but all the analyzed ones have at least one for NTR (Florencio et al., 2006; Balsera et al., 2014). This could suggest a prevalent role in thioredoxin reduction by the NTR over the FTR, but the cyanobacteria lacking FTR correspond almost to the genera *Prochlorococcus* strains that live in the oceans, representing a stable environment (García-Fernández et al., 2004; Dufresne et al., 2005; Florencio et al., 2006). NTRs in cyanobacteria are more diverse than in photosynthetic eukaryotes. At least three different phylogenetic groups are distinguishable in cyanobacteria, including NTRC (Florencio et al., 2006). However, not all cyanobacterial species contain a gene coding for NTRC. It has been proposed that there are, at least, two different strategies to cope with ROS in cyanobacteria, one involving the NTRC system and a second one consisting on a high peroxidase/catalase activity, such as described for *Synechocystis* (Pascual et al., 2010, 2011). *Anabaena* has been predicted to contain three *ntr* genes in its genome, with *all0737* being the one corresponding to the AnNTRC. Interestingly, the gene *alr2204*, coding for another NTR in *Anabaena*, is next to a gene for a Trx (*alr2205*), and it has been proposed that the *ntrC* gene in fact resulted from a fusion of these two genes. For instance, both *ntr* genes corresponding to *all0737 and alr2204* are grouped together by phylogenetic analysis (Florencio et al., 2006).

Until now, the cyanobacterial NTR has not been deeply studied. Only four of these proteins, predicted to be NTR by sequence analysis, have been demonstrated to have actual thioredoxin reductase activity. There are two reports about the *Synechocystis* NTR (Hishiya et al., 2008; Marteyn et al., 2009) concluding that this enzyme is involved in antioxidant and selenate toxicity protection, and that it is able to perform glutarredoxin reduction. Regarding NTRC, two of them have been studied in cyanobacteria (Sueoka et al., 2009; Pascual et al., 2011). The one from *Thermosynechococcus elongatus* was co-purified with a 2-Cys Prx, suggesting an *in vivo* interaction, and the system was reconstituted *in vitro* (Sueoka et al., 2009). The same interaction was described for the *Anabaena* NTRC when it was biochemically compared with the plant enzyme, showing that the cyanobacterial protein is able to partially replace the plant enzyme in *Arabidopsis* mutants (Pascual et al., 2011). In addition, indirect indications showed the *in vivo* 2-Cys Prx reduction by AnNTRC in *Anabaena* (Pascual et al., 2010). However, the lack of direct *in vivo* data about cyanobacterial NTRC was hindering the understanding of the endogenous function of this enzyme. Therefore, it was necessary to generate an *Anabaena* strain lacking the *ntrC* gene to further analyze its role *in vivo* and specially its involvement in the antioxidant defense system.

After verifying that the ΔntrC mutant strain was perfectly viable we subjected it to three different oxidative stress treatments. In all cases, the ΔntrC mutant displayed a higher sensitivity to the treatment, although the strongest effect was observed when the cyanobacterium was treated with H_2_O_2_. In the case of MV or HL treatments the differences with respect to the WT strain were lower. As it has been described before, both MV and HL primarily resulted in the production of superoxide anions that could later be transformed into hydrogen peroxide by the concourse of the superoxide dismutase enzyme (SOD activity). However, the amount of H_2_O_2_ produced in this way is lower than compared to direct external addition of H_2_O_2_ to the cell culture. Our results suggest that AnNTRC participates as component of the antioxidant protection system and, more specifically, in hydrogen peroxide detoxification, confirming the same role for this protein in cyanobacteria and plants (Serrato et al., 2004; Pérez-Ruiz et al., 2006).

It has been previously reported for plants, and *in vitro* for cyanobacteria, that once 2-Cys Prx reduces hydrogen peroxide, it is regenerated to its reduced form by NTRC (Kirchsteiger et al., 2009, 2012; Sueoka et al., 2009). The 2-Cys Prxs have been described as a component of the antioxidant response in plants and cyanobacteria (Klughammer et al., 1998; Yamamoto et al., 1999; Dietz et al., 2002; Pascual et al., 2010; Puerto-Galán et al., 2013). In cyanobacteria this Prx is overexpressed upon oxidative stress conditions (Pérez-Pérez et al., 2009) and it gets overoxidized in the presence of high peroxide concentrations (Pascual et al., 2010), what makes it a good indicator for intracellular oxidative stress in cyanobacteria. Here, we have checked both expression and overoxidation of 2-Cys Prx during the different treatments applied to the *Anabaena* WT and mutant strains. We could detect the monomer species during the three treatments (**Figures 3C, 4C**, and **5C**) and, since the overoxidation of the Prx is peroxide-dependent, the effect was more dramatic when H_2_O_2_ was added (**Figure 3C**). The overoxidation was more prolonged in the ΔntrC mutant due to the decrease in the electron transfer to the 2-Cys Prx. Although, it was shown for *Synechocystis* that the reduction of this enzyme could be performed by other Trxs (Pérez-Pérez et al., 2009) we could speculate that AnNTRC is the main electron donor in *Anabaena*, with the other Trxs partially taking over this role. Without this take-over, AnNTRC would be essential and the mutant would not be viable under oxidative stress conditions and the 2-Cys Prx would be completely overoxidized. However, further investigation is needed to demonstrate this.

Another enzyme, Srx, is responsible of reducing the 2-Cys Prx from the overoxidized state and, together with AnNTRC and this Prx, it constitutes the peroxide detoxification system in *Anabaena* (Pascual et al., 2010; Boileau et al., 2011). In this regard, an *Anabaena* mutant lacking the Srx protein becomes more sensitive to oxidative stress and the 2-Cys Prx appears strongly overoxidized and inactive, which results in an increase in intracellular ROS (Boileau et al., 2011). This effect is quite similar to what we state here, proving the importance of this system in oxidative stress response. Using the ROS-detecting probe DCFH-DA (**Figure 2B**) we show that there is an increase in ROS even under normal growth conditions, suggesting that when the AnNTRC is deleted the cyanobacterium has difficulties to deal with the normal cell ROS production associated to photosynthetic operation. This is also supported by the fact that the expression of the different genes selected as reporters for oxidative stress is higher in the mutant strain compared with the WT even before the treatments (**Figures 2C, 3D, 4D**, and **5D**; Supplementary Figure S1). In addition, we can see how the expression of the *2cysprx* gene is higher in the WT upon H_2_O_2_ treatment, which could be explained as an effort of the cyanobacterium to compensate the less efficient peroxide detoxification by the AnNTRC-Prx system. Under the other two treatments, the expression level of the reporter genes are lower in the case of the WT strain (**Figures 3D**, and **4D, 5D**; Supplementary Figure S1), pointing at the same direction of a higher oxidative stress in the mutant strain. Therefore, all these results demonstrate a role for AnNTRC in antioxidative response and peroxide detoxification in the cyanobacterium *Anabaena*. This idea is also supported by the fact that the gene and protein expression of this enzyme is increased upon the three oxidative stresses tested (**Figures 3–5**), resulting into a higher NTR activity in the cell, as was tested for the MV treatment (Supplementary Figure S2).

In the last part of our work we wanted to check if the AnNTRC also performs a new chaperone activity recently reported in *Arabidopsis* involved in thermotolerance (Chae et al., 2013). As it was described in plants, the *Anabaena* mutant strain was more sensitive to heat shock treatments (**Figure 6** and Supplementary Figure S3) and we were able to detect HMM complexes of AnNTRC when high temperature was applied (**Figure 7**). These complexes are thiol-dependent since under reducing conditions they are not observable (**Figure 7A**). However, further analyses are needed in order to study the chaperone activity of these complexes in cyanobacteria. In addition, it would be necessary to unravel if AnNTRC and 2-Cys Prx are also linked in this new role since chaperone activities have been also described for HMM complexes of this kind of Prxs (Morgan and Veal, 2007; Barranco-Medina et al., 2009; Dietz and Pfannschmidt, 2011).

## Author Contributions

FF designed research, CG and AM-C constructed the NTRC mutant, AS-R analyzed the NTRC mutant and carried out the molecular characterization of the mutant and the oxidative stress treatments. FF, AM-C and AS-R performed the data analysis. AM-C, AS-R, and FF wrote the paper.

## Conflict of Interest Statement

The authors declare that the research was conducted in the absence of any commercial or financial relationships that could be construed as a potential conflict of interest.

## Abbreviations

FTR: Ferredoxin-thioredoxin reductase
HL: high light
HMM: high-molecular mass
MV: methyl viologen
NTR: NADPH-thioredoxin Reductase
Prx/s: peroxiredoxin/s
ROS: reactive oxygen species
SEC: size exclusion chromatography
SOD: superoxide dismutase
Srx: sulfiredoxin
Trx/s: thioredoxin/s
