# Supplementary material for: NADPH-Thioredoxin Reductase C Mediates the Response to Oxidative Stress and Thermotolerance in the Cyanobacterium Anabaena sp. PCC7120
Source: Front Microbiol. 2016 Aug 18;7:1283. doi: 10.3389/fmicb.2016.01283 (PMC4988983; doi:10.3389/fmicb.2016.01283)
Supplement: Supplementary file 1 [file Data_Sheet_1.PDF]

**NtrC_Up_F**	GTAATAGCCTCGAGCTTTCCATATCG
**NtrC_Up_R**	GCGGATCCTTCAAATACAACGGGTTTTAGGTTAGC
**NtrC_Down_F**	AAGGATCCGCTAAAGTGGTTGATGAATTTGACAGC
**NtrC_Down_R**	GCGTCCACCTGCAGGGTCATCTGCTC
**NtrC_F3**	GTCGGGGAATTTCGGCTTG
**NtrC_R3**	CTACCAGCCGCCGTAATTGC
**An2CysPrx_F**	CAACAGCTGTAGTTGATCAGG
**An2CysPrx_R**	CTTCATCTGGGTGAGACTGG
**AnPrxII_F**	GGTCAAAGAGTTCCTCAAGTC
**AnPrxII_R**	GTTGTTGCACCCGTGCACGC
**AnSrxA_F1**	GGTTAGGGTACAAGAAATTC
**AnSrxA_R1**	CTACGCTTAGTGCATC
**AnIsiA_F1**	CGCTTCGCCAATCTCTCTG
**AnIsiA_R1**	AACCTGAGTTGTTGCGTCG
**AnrnpB_F**	GCAGACCAGTTAGCTTAACTG
**AnrnpB_R**	CTTTACACGAGGGCGATTATC

Table S1: Primers used in this study.

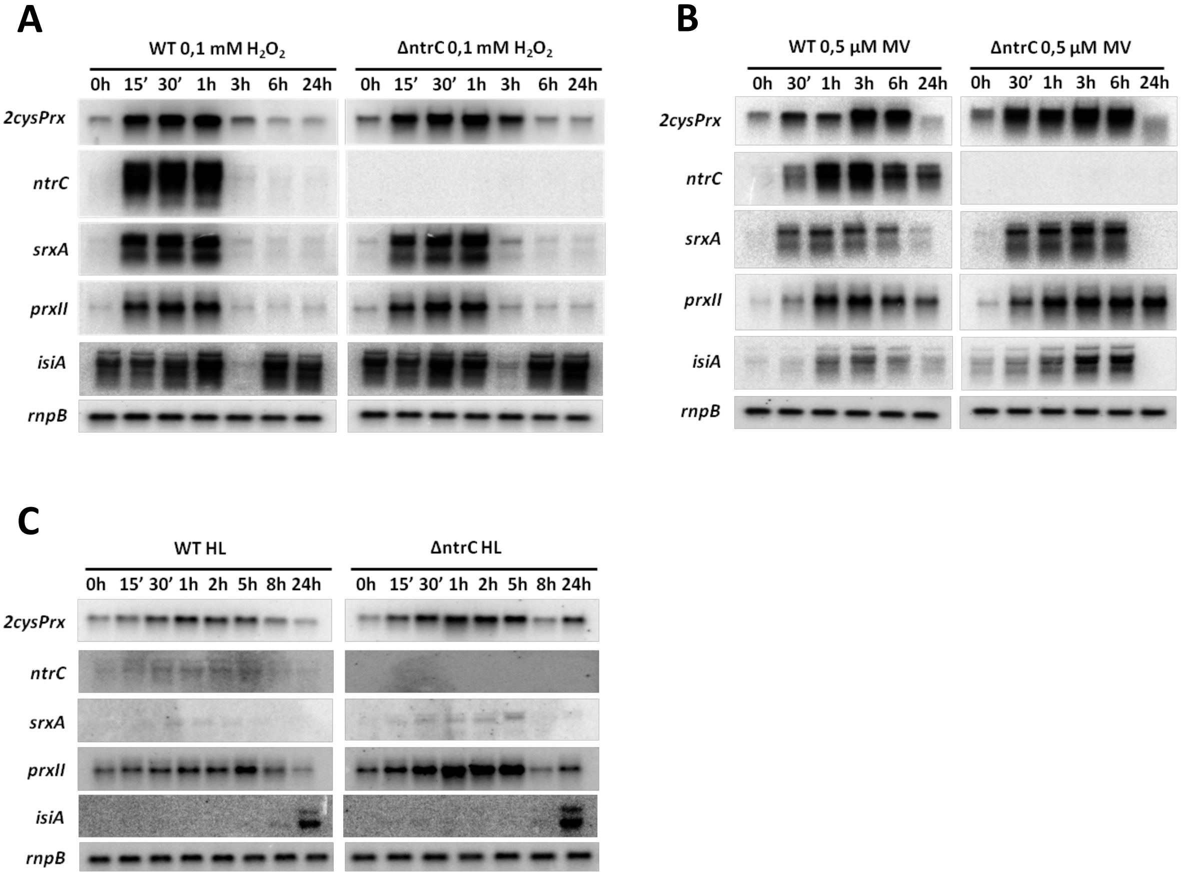

Figure S1: Northern blot analysis of *2cysprx*, *ntrC*, *srxA*, *prxII* and *isiA* expression in response to H_2O_2 (A), MV (B) and High Light (C) treatments. Total RNA was isolated from WT cells grown in BG11C at the indicated times after a 0,1 mM H_2O_2, 0,5 µM MV additions or the shift from 50 to 500 $\mu E \cdot m^{-2} \cdot s^{-1}$ light intensity. The filters were hybridized with *2cysprx, ntrC*, *srxA, prxII* and *isiA* probes and subsequently stripped and re-hybridized with an *rnpB* probe as a control.

A

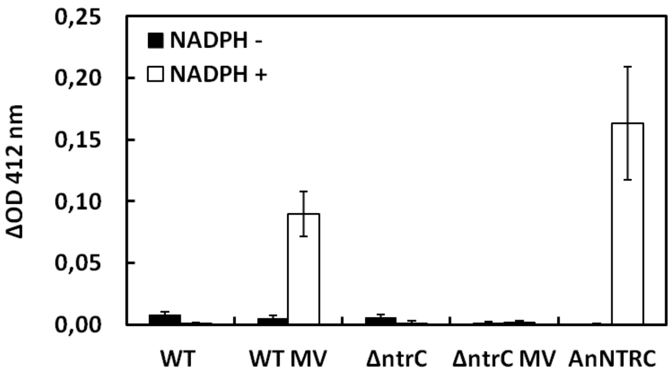

B

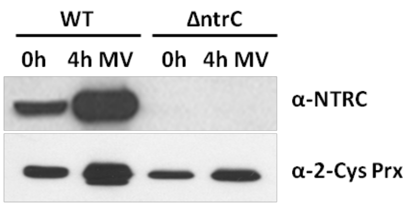

Figure S2: DTNB reductase activity of AnNTRC. A. NADPH-dependent reduction of DTNB was assayed in crude extracts from WT and ΔntrC culture cells treated or not with 0,5 µM MV for 4 h. Purified AnNTRC was also used as a control. Assays were performed at least three times and error bars represent SE. **B.** Western blot analysis of AnNTRC and 2-Cys Prx in response to MV. WT and ΔntrC strains cells grown in BG11C and samples were collected at the indicated time after 0,5 µM MV addition. Five micrograms of total protein from soluble extracts were separated by 12% SDS-PAGE and subjected to Western blot to detect AnNTRC and 2-Cys Prx.

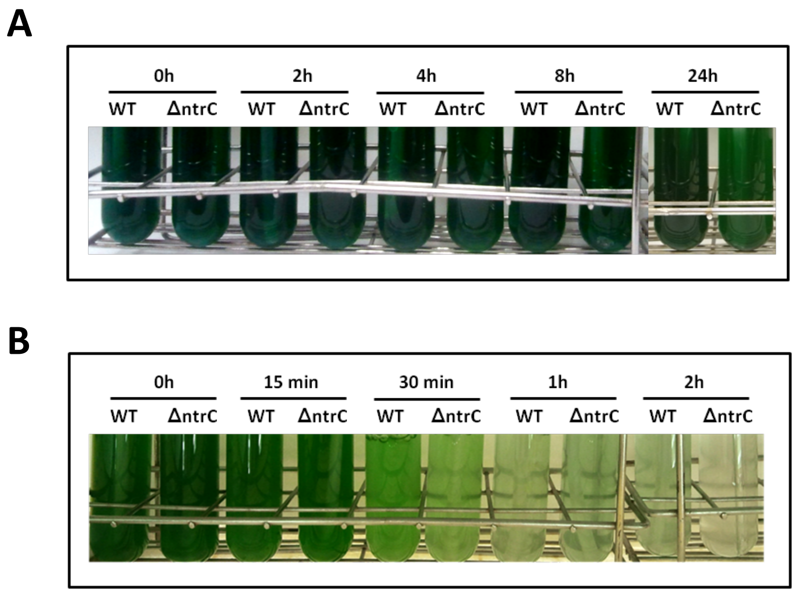

Figure S3: Growth recovery after heat shock. Pictures of the cultures recovered by growing 24 h at 30 °C previously subjected to high temperature treatment during the indicated times at 45 °C **(A)** and 50 °C **(B)**. The pictures correspond to a representative replicate of the experiment depicted in Figure 6 B (45 °C) and D (50 °C).
